# SAMHD1 expression is a surrogate marker of immune infiltration and determines prognosis after neoadjuvant chemotherapy in early breast cancer

**DOI:** 10.1007/s13402-023-00862-1

**Published:** 2023-09-04

**Authors:** Lucía Gutiérrez-Chamorro, Eudald Felip, Eva Castellà, Vanessa Quiroga, Ifeanyi Jude Ezeonwumelu, Laura Angelats, Anna Esteve, Laia Perez-Roca, Anna Martínez-Cardús, Pedro Luis Fernandez, Angelica Ferrando-Díez, Anna Pous, Milana Bergamino, Beatriz Cirauqui, Marga Romeo, Iris Teruel, Ricard Mesia, Bonaventura Clotet, Eva Riveira-Muñoz, Mireia Margelí, Ester Ballana

**Affiliations:** 1 AIDS Research Institute-IrsiCaixa and Health Research Institute Germans Trias i Pujol (IGTP), Hospital Germans Trias i Pujol, Universitat Autònoma de Barcelona, 08916 Badalona, Spain; 2 Medical Oncology Department, Catalan Institut of Oncology (ICO)-Badalona, B-ARGO (Badalona Applied Research Group in Oncology) and IGTP (Health Research Institute Germans Trias i Pujol), Universitat Autònoma de Barcelona, 08916 Badalona, Spain; 3 Department of Pathology, Hospital Germans Trias i Pujol, IGTP (Health Research Institute Germans Trias I Pujol), Universitat Autònoma de Barcelona, 08916 Badalona, Spain; 4 Banc de Tumors, Health Research Institute Germans Trias i Pujol (IGTP), Hospital Germans Trias i Pujol, Universitat Autònoma de Barcelona, 08916 Badalona, Spain

**Keywords:** SAMHD1, Immune infiltration, TCP1 complex, IL12-family signaling

## Abstract

**Purpose:**

The lack of validated surrogate biomarkers is still an unmet clinical need in the management of early breast cancer cases that do not achieve complete pathological response after neoadjuvant chemotherapy (NACT). Here, we describe and validate the use of SAMHD1 expression as a prognostic biomarker in residual disease *in vivo* and *in vitro*.

**Methods:**

SAMHD1 expression was evaluated in a clinical cohort of early breast cancer patients with stage II-III treated with NACT. Heterotypic 3D cultures including tumor and immune cells were used to investigate the molecular mechanisms responsible of SAMHD1 depletion through whole transcriptomic profiling, immune infiltration capacity and subsequent delineation of dysregulated immune signaling pathways.

**Results:**

SAMHD1 expression was associated to increased risk of recurrence and higher Ki67 levels in post-NACT tumor biopsies of breast cancer patients with residual disease. Survival analysis showed that SAMHD1-expressing tumors presented shorter time-to-progression and overall survival than SAMHD1 negative cases, suggesting that SAMHD1 expression is a relevant prognostic factor in breast cancer. Whole-transcriptomic profiling of SAMHD1-depleted tumors identified downregulation of IL-12 signaling pathway as the molecular mechanism determining breast cancer prognosis. The reduced interleukin signaling upon SAMHD1 depletion induced changes in immune cell infiltration capacity in 3D heterotypic *in vitro* culture models**,** confirming the role of the SAMHD1 as a regulator of breast cancer prognosis through the induction of changes in immune response and tumor microenvironment.

**Conclusion:**

SAMHD1 expression is a novel prognostic biomarker in early breast cancer that impacts immune-mediated signaling and differentially regulates inflammatory intra-tumoral response.

**Supplementary Information:**

The online version contains supplementary material available at 10.1007/s13402-023-00862-1.

## Introduction

Breast cancer (BC) is a heterogeneous disease that requires personalized treatment strategies. Neoadjuvant chemotherapy (NACT) has become part of the standard-of‑care treatment of patients with early stage breast cancer [1–4]. Numerous studies have demonstrated that achievement of a pathological complete response (pCR) after NACT increases both overall survival (OS) and disease-free survival (DFS) compared with patients with residual invasive disease, especially in triple-negative and estrogen receptor (ER) negative/HER2-positive patients [5–8]; while the benefits in ER-positive tumors are less clear [9–11]. Despite the evident benefits and increased use of NACT, there is still a clinical need for the identification of prognostic biomarkers of response in residual disease. Characterization of residual disease post NACT should have a strong influence on patient prognosis. Remarkably, NACT affects both tumor cells and their microenvironment, and biomarkers to guide the choice of an adjuvant therapy might be found in either compartment. However, at present, prognostic biomarkers based on post-NACT breast-cancer characteristics remain scarce and more indicators of a patient’s long-term prognosis are needed to help personalize subsequent adjuvant treatments [12]. Currently the proposed biomarkers include (i) classic histopathological parameters (Ki67 expression, grade and lymph vascular invasion); (ii) genomic and proteomic markers (gene expression, mutation profile, circulating tumoral DNA) and (iii) immune-based markers, which are among the most-promising in the post NACT setting.

The importance of biomarkers related to the tumor microenvironment (TME) is stemming from numerous discoveries revealing that cellular and acellular components of the TME can promote or suppress tumorigenesis[13]. In addition, growing evidence supports a role of the TME in the modulation of tumor responses to therapy, although currently, immunotherapy response in breast cancer is heterogeneous and limited to triple negative tumors [14, 15]. Therefore, much more work is needed to optimize patient selection based on immune-based biomarkers; being fundamental the in-depth characterization of the dynamic interaction between the tumor and the immune cells [16]. Indeed, in BC, an elevated immune infiltration with a greater diversity in the response of T cells has been associated with a better outcome and with better survival in HER2-negative patients, particularly when the infiltrate is mainly CD8 + T cells [17, 18]. In addition, high immune infiltration of T cells has been related to an increase in the response to NACT [19] and with a decrease in tumor proliferation measured as a reduction in intratumorally Ki67 [20]. In contrast, increased levels of Tregs and myeloid-derived suppressor cells (MDSCs), two immunosuppressive cell populations important in the establishment and maintenance of cancer immune tolerance, has been reported to be associated with a poor response to NACT and a poor clinical outcome in BC patients [21–23].

Recently, the human sterile alpha motif and HD-domain-containing protein 1 (SAMHD1) has been linked to cancer onset and progression, as well as to chemosensitivity to distinct anticancer drugs, with numerous and controversial evidences [24–27]. SAMHD1 is the only deoxynucleotide triphosphohydrolase (dNTPase) in eukaryotes and beyond cancer, it has been linked to other pathological processes, including viral restriction, modulation of immune response and autoimmune diseases. Genetic mutations in SAMHD1 causes the Aicardi-Goutières Syndrome [28], a rare genetic disorder characterized by excessive interferon (IFN) production resulting in systemic inflammatory injury [29, 30], that affects the brain, spinal cord and immune system. Previous data from our group and others suggested that SAMHD1 determines prognosis and treatment efficacy in different solid tumor types, including BC [30]. These observations together with the reported role of SAMHD1 as a modulator of the immune response prompted us to test the prognostic value of SAMHD1 in residual disease after surgery in a series of patients with early BC treated with NACT. Moreover, the molecular mechanisms underlying SAMHD1 function in breast tumors have also been studied in T47D tumor spheroids, leading to specifically delineate the immunomodulatory consequences of SAMHD1 knockout, *in vitro* and *in vivo* through the dysfunction of specific interleukin signaling pathways.

## Materials and methods

### Patients and samples

**Fig. 1:**
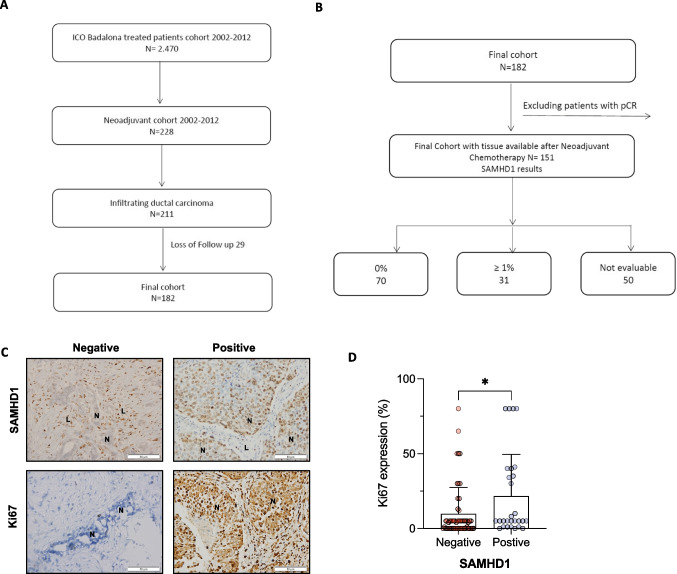
SAMHD1 is variably expressed in breast cancer tumors and correlates with Ki67 expression. **A** Flow chart of the BC cohort of patients included in the study. **B** Flow chart of the patients used to analyze SAMHD1 expression and SAMHD1 results. Of 182 patients originally included, only 151 had sufficient tumor tissue to perform SAMHD1 assessment. **C** Expression of SAMHD1 and Ki67 by immunochemistry in tumor samples. Representative microscopy images of SAMHD1 (upper panel) and Ki67 (lower panel) expression in paraffin-embedded tumor biopsies. Only tumor cell expression of SAMHD1 and ki67 was considered. High expression of SAMHD1 was observed in lymphocytic cells infiltrating in the tumors, and it was used as a positive control of immunohistochemistry for negative biopsies. N, neoplastic cells; L, lymphocytes. SAMHD1 expression was exclusively nuclear. **D** Percentage of Ki67 expression was significantly lower in SAMHD1-negative tumors post-NACT

We collected tumor biopsies from 182 patients from ICO Badalona diagnosed with stage II and III early BC between 2002 and 2012. Included patients were not eligible for conservative breast treatment and represented candidates for primary therapy according to the decision of the Breast Cancer Tumor Board of our institution (Fig. 1A). The study was conducted in accordance with the ethics principles of the Declaration of Helsinki and approved by the Research and Ethics Committee of Hospital Germans Trias i Pujol. Samples were obtained from the Biobank of the Institut d’Investigació Germans Trias i Pujol. All patients provided written informed consent.

All included patients received NACT with anthracycline-based regimens with or without taxanes. In patients with HER2 amplification, trastuzumab was added to taxanes-based chemotherapy. After surgery, patients were evaluated and received additional therapy according to the anatomopathological result as follows, trastuzumab in HER2 + cases and adjuvant hormone therapy in estrogen receptor (ER) and/or and progesterone receptor (PR) positive cases. Patients undergoing lumpectomy also received breast radiotherapy and regional nodal radiation at the discretion of the breast cancer committee. Patients without residual infiltrating disease in the breast and axilla were considered to have had a pathological complete response (pCR). Supplementary Table 1 summarizes patients characteristics of the cohort. Tumor stage was classified according to the TNM classification of the Union International Cancer Control [31]. Treatments were obtained from the review of medical records.

### Construction of tissue microarrays (TMA) and immunohistochemical methods

TMA were prepared by extracting two 1 mm diameter cores of histologically confirmed invasive breast carcinoma using a TMA workstation MTA-1 (Beecher instruments). Two cores/ tumor were evaluated and scored and the mean of the two readings was calculated. TMA were cut in 5 µm sections and subsequently stained for ER, PR, basal cytokeratins, HER2 and Ki67 following standardized methods used for routine clinical testing. TMA were also stained for SAMHD1 expression (polyclonal rabbit anti-SAMHD1 antibody, cat. no. 12586–1-AP, Proteintech), using an automated detection system (Ultra view, Ventana 9 after antigen retrieval), as previously reported [30]. The percentage of SAMHD1 stained cells was evaluated only in tumoral cells and was measured as positive or negative, defining SAMHD1 positive cases as those with cellular positivity ≥ 1%. Expression of SAMHD1 in other cell types from TME was only considered in negative cases, as high expression of SAMHD1 is well characterized in all immune cells. All immunohistochemical analyses were performed in the histopathological unit of Hospital Germans Trias i Pujol.

### Cells and cell culture

Human T47D cells and MCF7 cells were obtained from Sigma-Aldrich-ECACC (European Collection of Authenticated cell cultures, 85102201-1VL) and grown in complete Dulbecco's modified Eagle's medium (DMEM, cat num. 11965-092,ThermoFischer) supplemented with 10% of heat-inactivated fetal bovine serum (FBS, ThermoFisher) and antibiotics 100 U/ml penicillin, 100 μg/ml streptomycin (Life Technologies) and maintained at 37°C in a 5% CO_2_ incubator.

Peripheral blood mononuclear cells (PBMCs) were obtained from blood of healthy donors using Ficoll-Paque density gradient centrifugation. Total PBMCs were cultured in RPMI media supplemented with 10% heat-inactivated FBS (cat. num. 11875093, ThermoFisher) and 100 U/ml penicillin, 100 μg/ml streptomycin (Life Technologies).

### Generation and evaluation of SAMHD1 *knock-out* cells

For generation of *knock-out* (KO) cells, T47D cells were transfected with a plasmid expressing a CRISPR-Cas9 construct designed to disrupt the sequence corresponding to exon 5 of *SAMHD1* gene that encodes for HD domain (CRISPR-SAMHD1), as described previously [32]. Cell proliferation was evaluated by growth curves. Cells were plated in 96-well plates at 5000 cells/well. At indicated time points, cells were lysed with CellTiter-96® AQ_ueous_ One Solution Reagent (cat num. G3582, Promega) and luminescence was read using the Envision plate reader. Sensitivity to NACT in SAMHD1-KO cells was measured by the tetrazolium-based colorimetric method (MTT) as described elsewhere [33]. Cells were treated at indicated doses of doxorubicin and/or paclitaxel (cat num. D2975000 and Y0000698, Sigma-Aldrich) for 4 days.

### Generation of SAMHD1 *knock-down* cells by RNA interference (RNAi)

For SAMHD1 *knock-down* in MCF7 cells, siRNAs targeting *SAMHD1* gene (siSAMHD1) and non-targeting control (siNT) (siGENOME SMARTpool; Dharmacon, Cultek) were transfected following standard procedures. In brief, 100 nM of the corresponding siRNA were mixed with Lipofectamine 3000 reagent (cat num. L3000001, ThermoFisher) and added to 200,000 MCF7 cells seeded in 24-well plates in Optimem medium without FBS. After 24h, complete DMEM was added and SAMHD1 depletion evaluated by RNA and protein expression.

### Generation of breast cancer spheroids and establishment of immune cell-tumor cocultures

T47D or MCF7 spheroids were generated by seeding 5 × 10^3^ cells per well on Nunclon Sphera round bottom plates (cat num. 174925, ThermoFisher) in RPMI medium. The following day, complete RPMI medium was added, and spheroids were allowed to grow for 3 more days. Immune cell-tumor co-cultures were established 4 days after spheroid formation by adding 5 × 10^5^ PBMCs per spheroid and well. Cell aggregation was excluded by extensive washing of cocultures before processing the samples for flow cytometry or immunofluorescence.

### RNA-Sequencing and library preparation

Cellular RNA was extracted using the NucleoSpin RNA II kit (cat num. 740955, Magerey-Nagel), as recommended by the manufacturer, including the DNase I treatment step. RNA-sequencing samples were prepared in biological duplicates and from two distinct SAMHD1 KO T47D cell clones, representing 4 distinct replicates of SAMHD1 KO. After quality control check, RNA library was constructed using Illumina TruSeq Stranded mRNA LT Sample Prep Kit and sequencing was performed using NovaSeq 6000 System with 150 bp paired ends reads (Macrogen).

### Transcriptomic analysis

Transcriptomic analysis was performed as implemented in the computational workflow for the detection of differentially expressed genes and pathways from RNA-seq data [34]. Reads were aligned to the human GRCh38 (annotation NCBI_109.20200522) using HISAT2. Low-expression genes with at least one zero counts were filtered out and the remaining reads normalized with Relative Log Expression (RLE) method as implemented in DESeq2 R library. Differential gene expression between the control and treatment groups was estimated with the DESeq2 Wald test. Sequencing files can be accessed on gene expression omnibus repository (GSE224678).

Gene set enrichment analysis (GSEA) was performed on the preranked (based on log2FC) differentially expressed gene dataset derived from the WT vs. SAMHD1-KO comparison. Fast pre-ranked gene set enrichment analysis (GSEA) was implemented with the fgsea R package [35], by setting the estimated p-value argument to zero in order to estimate the p-value more accurately. As gene set database files in GSEA analyses, we used the hallmark (h.all.v7.5.1.symbols.gmt) and Reactome (c2.cp.reactome.v2022.1.Hs.symbols.gmt) signature files in gmt format retrieved from the Molecular Signatures Database (https://www.gsea-msigdb.org/gsea/msigdb/, accessed on 16 September 2022 and 2 October 2022, respectively) [36]. Weighted enrichment statistics were based on 1000 permutations. The normalized enrichment score (NES) reflects the degree to which a gene set-signature-pathway was overrepresented at the extremes (top-up-regulated or bottom-down-regulated) of the entire ranked list of differentially expressed genes (DEGs) by the comparison of WT vs. KO cells. The score was calculated by walking down the list of DE genes, increasing a running-sum statistic when we encountered a gene in our gene signature and decreasing it when we encountered genes not included in our gene signature. The enrichment score depicts the maximum deviation from zero encountered in the random walk. Significantly enriched gene-sets with FDR adjusted q-value < 0.1 were selected for Enrichment map visualization. For the visualisation of the differentially altered pathways, customised bar plots were created using the Cytoscape program [37]. Briefly, enrichment files were inputted into the Enrichment Map app within the Cytoscape program for visualization. Parameters were set at default values (node cutoff FDR Q value 0.1; Jaccard Overlap combined coefficient cutoff 0.375, k-constant 0.5). Nodes were manually laid out and combined into a common biological process for clarity using the AutoAnnotate app (v1.4.0, https://apps.cytoscape.org/apps/autoannotate).

### Quantitative RT-polymerase chain reaction (qRT-PCR)

RNA from tumor samples was extracted from six 10 μm slides per tumor using the RNeasy FFPE Kit (cat num. 73504, Qiagen), following manufacturer recommendations. The protocol included deparaffinization with xylene and enzymatic processing with proteinase K and DNAase. The resulting RNA was quantified using the Nanodrop and only samples with a minimum concentration of 50ug/ul and a 260/280 absorbance ratio between 1.7 and 2.3, were used. For relative cell mRNA quantification, RNA was extracted using the NucleoSpin RNA II kit (Magerey-Nagel), as described above.

Reverse transcriptase was performed using the PrimeScript™ RT-PCR Kit (cat num. RR014B, Takara). mRNA relative levels of all genes were measured by two-step quantitative RT-PCR and normalized to *GAPDH* mRNA expression using the DDCt method, using the following TaqMan gene expression assays (*IL6*, TaqMan Hs00174131_m1; *SAMHD1*, TaqMan Hs00174103_m1; *IL8*, TaqMan Hs00174103_m1; *IL1α*, TaqMan Hs00174092_m1; *CRLF1*, TaqMan Hs00191064_m1; *CNN2* TaqMan Hs04377468_g1; *TCP1* TaqMan Hs01053946_g1; *TYK2* TaqMan Hs01105947_g1; *GAPDH* TaqMan Hs00266705_g1, all from ThermoFisher).

### Quantification of IL6 and IL8 proteins

IL6 and IL8 quantification in culture supernatants were determined using ProQuantum immunoassays, following manufacturer’s protocol (cat num. A35573 and A35575, Thermofischer). Each sample was assayed in duplicate.

### Western blot analysis

Cells were rinsed in ice-cold PBS, extracts were prepared in lysis buffer (50 mM Tris HCl pH 7.5, 1 mM EDTA, 1 mM EGTA, 1 mM NaV_3_O_4_, 10 mM sodium β-glycerophosphate, 50 mM NaF, 5 mM sodium pyrophosphate, 270 mM sucrose and 1% Triton X-100) supplemented with protease inhibitor cocktail (cat num. 11836153001, Roche) and 1 mM phenylmethylsulfonyl fluoride. Samples were electrophoresed in SDS–polyacrylamide gels and blotted onto nitrocellulose membranes. Blocked membranes were incubated overnight at 4 °C with the following antibodies: anti-human GAPDH (1:10000, ab9485, Abcam); anti-Cleaved PARP1 [E51] (1:1000, ab32064, Abcam) and anti-cleaved caspase 3 (1:10000, ab9485, Abcam) and anti-γH2AX (Ser139) (1:1000, ab2577, Cell Signaling). After washing, the membranes were incubated with a secondary conjugated horseradish peroxidase antibody for 1 h at room temperature and then revealed with SuperSignal West Pico Chemiluminescent Substrate (cat num. 34580, PierceChemical).

### Immunophenotypic characterization of PBMCs by flow cytometry

Cocultures were isolated 3 days after establishment. After pooling 6 co-cultures, breast cancer spheroids were extensively washed and trypsinized to obtain a cell suspension. Fc receptors were blocked with Human TruStain FcX™ (Fc Receptor Blocking Solution, cat num. 422301, BioLegend) for 10 min at RT, prior to incubation with the following antibodies: CD3-APC-Cy7, CD4-APC, CD14-BV510, CD15-BV650, CD16-PerCP-Cy5.5, CD56-Pe-Cy7 and CD86-BV786 (all from BioLegend) for 30 min at 4°C in the dark. Cells were washed and fixed in 1% formaldehyde prior to analyzing in the flow cytometer (FACS LSRII, BD Biosciences). Immunophenotyping of different cell subsets were defined based on described expression combinations gated on the live singlet lymphocytes. Data was analyzed using the FlowJo software (BD Biosciences).

### Imaging

T47D cells were pre-stained before T47D spheroid formation with CellTracker™ Red CMTPX Dye (cat num. C34552, ThermoFisher) and PBMCs prior to co-culture establishment with CellTracker™ Green CMFDA Dye (cat num. C7025, ThermoFisher), according to manufacturer procedure. Cocultures were left for 3 days, pooled and washed as described above, prior to embedding in Epredia™ HistoGel™ Specimen Processing Gel (cat num. HG-4000–012, ThermoFisher). Afterwards, samples were included in paraffin and cut in 5 μm-thick sections. The slides were mounted with DAPI-containing Fluoromount-G (cat num. 0100–01, ThermoFisher) and imaged using a fluorescence microscope (Nikon Ts2R-FL). Quantification of CFSE + cells infiltrating the T47D spheroids was done using the ImageJ software.

### Statistical analysis

A comprehensive cohort description analysis based on demographic, clinical and biological data was performed. Categorical variables were summarized through frequencies and percentages and quantitative variables using means and standard errors or medians and interquartile ranges. Time to progression (TTP) was defined as the time from date of cohort treatment initiation to date of progression and Overall survival (OS) was defined as the time from date of cohort treatment initiation to date of death resulting from any cause. Patients who were alive (for OS) or disease free (for TTP) will be censored at the date of last follow-up. Median times for TTP, OS, will be estimated using the method of Kaplan–Meier and reported with their confidence intervals (CI) at the 95% level. Log-rank test was used to compare survival curves between groups of patients. Univariate and multivariate Cox regression models were performed to analyze variables associated with the risk of progression or death. Hazard ratios (HRs) and 95% confidence intervals were reported.

Experimental data were analyzed with the PRISM statistical package. If not stated otherwise, all data were normally distributed and expressed as mean ± SD. *p*-values were calculated using an unpaired, two-tailed, t-student test. Statistical significance for *in vitro* and *ex vivo* experiments was calculated using appropriate t-test in GraphPad Prism (v9.3.0). All experiments were performed in at least three independent replicates and n values are provided in the figure legends. Analysis were performed using GraphPad Prism and R software.

## Results

### SAMHD1 expression is associated to increased risk of recurrence in post-NACT tumor biopsies

To evaluate the contribution of SAMHD1 expression in cancer progression and/or treatment, a retrospective study including all breast cancer patients treated in ICO Badalona during a 10-year period (2002–2012) was performed (*n* = 2470). Within the whole cohort, 11% of cases received NACT (*n* = 228) and SAMHD1 expression was evaluated in all infiltrating ductal carcinoma cases with follow-up (*n* = 182) that do not achieve pCR (*n* = 151, Fig. 1A). No significant clinical differences were found between the entire NACT cohort and the subgroup of patients without pCR (Supplementary Table 1). Expression of SAMHD1 was evaluated by immunohistochemistry in available retrospectively collected post-NACT tumor biopsies and samples were stratified according to SAMHD1 expression (Fig. 1B and C, upper panel). Residual tumors were SAMHD1 positive in 30% of evaluable patients (31/101 evaluable tumors).

Then, a comprehensive clinical description of the post-NACT cohort, stratified according SAMHD1 expression was performed (Table 1). No major differences were observed between SAMHD1 positive and negative tumors post-NACT, except for histological grade, Ki67 expression and recurrence. Interestingly, SAMHD1 positivity was associated with grade III tumors (*p* = 0.025), higher levels of Ki67 after NACT (*p* = 0.017) and increased risk of recurrence (p = 0.005), suggesting that SAMHD1 expression might represent a relevant prognostic biomarker for breast cancer post-NACT (Table 1, Fig. 1D).

**Table 1:** Clinical characterization of patients according to SAMHD1 expression in post-NACT tissue

Variable	SAMHD1 + (*n* = 31)	SAMHD1—(*n* = 70)	P-value
Age (y), Mean (IQR)	50.48 (11.09)	50.66(13.21)	.946
Menopausal status, n (%)			.274
Yes Not	16 (51.6%) 15(48.4%)	30 (42.9%) 40 (57.1%	
Clinical Stage			.864
IIA IIB IIIA IIIB IIIC	4 (12.9%) 4 (12.9%) 14 (45.2%) 9 (29%) 0 (0%)	11 (15.7%) 13 (18.6%) 27 (38.6% 18 (25.7%) 1 (1.4%)	
Clinical Stage			.273
II III	8 (25.8%) 23 (74.2%)	24 (34.3%) 46 (65.7%)	
Nodal status (Dg)			.213
Negative Positive	5 (16.1%) 26 (83.9%)	18 (25.7%) 52 (74.3%)	
Estrogen Receptor (ER)			.455
< 1 1–10 > 10	13 (41.9%) 3 (9.7%) 15 (48.4%)	21 (30%) 6 (8.6%) 43 (61.4%)	
Progesterone Receptor (PR)			.744
< 1 1–10 > 10	16 (51.6%) 1 (3.2%) 14 (45.2%)	35 (50%) 5 (7.1%) 30 (42.9%)	
HER2 (IHQ & FISH or CISH)			.286
Negative Positive	24 (77.4%) 7 (22.6%)	59 (84.3% 11 (15.7%)	
Ki67 Post surgery, mean (SD)	22.1 (28.32)	9.8 (17.59)	.**017**
Histological grade post-surgery			.069
I II III Unknown	3 (9.7%) 12 (38.7%) 15 (48.4%) 1 (3.2%)	10 (14.3%) 41 (58.6%) 19 (27.1%) 0 (0%)	
Histological grade post-surgery			**.025**
I and II III Unknown	15 (48.4%) 15 (48.4%) 1 (3.2%)	51 (72.9%) 19 (27.1%) 0 (0%)	
*Subtypes			.356
Luminal A Luminal B Luminal B HER2 positive HER2 positive Triple Negative	6 (19.4%) 9 (29%) 3 (9.7%) 4 (12.9%) 9 (29%)	23 (32.8%) 24 (34.3%) 2 (2.9%) 7 (10%) 14 (20%)	
Pre-surgery Chemotherapy			.709
Anthracyclines/taxanes + Trastuzumab Anthracycline based regimens Anthracycline and taxane based regimens	2 (6.5%) 13 (41.9%) 16 (51.6%)	3 (4.3%) 25 (35.7%) 42 (60%)	
Pathological Response			.310
PR ED PD	8 (25.8%) 22 (71%) 1 (3.2%)	17 (24.3%) 53 (75.7%) 0 (0%)	
Type of surgery			.390
Tumorectomy + AND Mastectomy + AND	9 (29%) 22 (71%)	18 (25.7%) 52 (74.3%)	
Complementary Chemotherapy			.428
Yes Not	11 (35.5%) 20 (64.5%)	22 (31.4%) 48 (68.6%)	
Complementary Hormonotherapy			.471
Yes Not	19 (61.3%) 12 (38.7%)	45 (64.3%) 25 (35.7%)	
Complementary Radiotherapy			.468
Yes Not	28 (90.3%) 3 (9.7%)	5 (7.1%) 65 (92.9%)	
Recurrence			**.005**
Yes Not	18 (58.1%) 13 (41.9%)	20 (28.6%) 50 (71.4%	
Type of relapse			.459
Only Local Distant (with or without local)	1 (5.5%) 17 (94.4%)	0 (0%) 20 (100%)	

^*^Luminal A: ER positive,PR > 20%, HER2 negative ki 67 < 14%; Luminal B: ER positive, PR < 20%, HER2 negative, and/or ki67 ≥ 14%; Luminal B HER2 positive: ER and/or PR positive, HER2 positive; HER2: HER2 positive;Triple Negative: ER negative, PR negative, HER2 negative.

In bold, statistically significant p-values

### SAMHD1 tumor positivity determines shorter TTP and OS after NACT

**Fig. 2:**
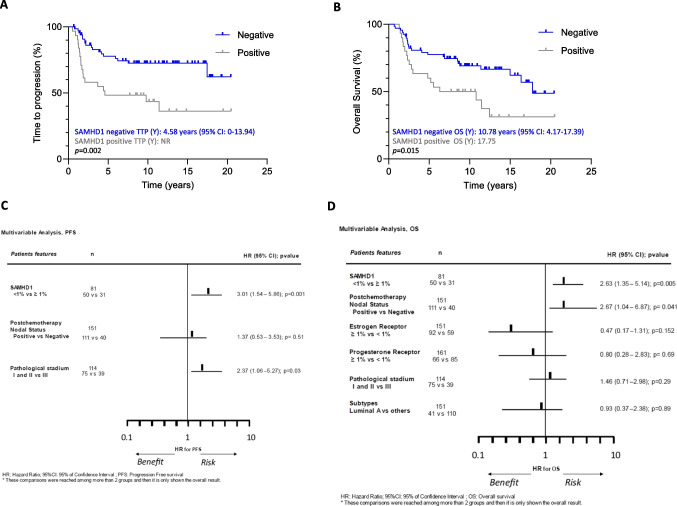
Prognostic value of SAMHD1 in breast cancer cohorts. Kaplan–Meier curves of time to progression (TTP) (**A**) and overall survival (OS) since treatment initiation/cancer diagnosis (**B**) according to SAMHD1 status. SAMHD1 negative patients after neoadjuvant chemotherapy have significantly better TTP and OS. SAMHD1 expression below 1% in cancer cells was considered as negative SAMHD1 (blue lines) and equal or above 1% was considered as positive SAMHD1 tumors (grey lines). Median survival times with CI 95% of both groups are shown. Log-rank test was used to compare survival curves between groups. Hazard ratios (HRs) and 95% confidence intervals (CIs) were obtained from the Cox multivariate model to analyze the risk of progression (**C**) and death (**D**). SAMHD1 negativity and pathological stadium I and II versus III after NACT, are associated with increased progression free survival; and SAMHD1 negativity and negative nodal affection are associated with overall survival improvement

To gain insight into the putative role of SAMHD1 as a prognostic factor in BC, TTP was evaluated. SAMHD1-expressing tumors presented shorter TTP than SAMHD1 negative cases (log-rank function, *p* = 0.002), with a median TTP of 4.58 years for SAMHD1-positive patients, whereas it was not reached for SAMHD1 negative patients (Fig. 2A). Similar results were obtained for OS, where median OS was significantly shorter for SAMHD1 positive tumors (10.78 years, 95%CI, 4.17–17.39 in SAMHD1 positive vs. 17.75 years, 95%CI, 0 to 14.6 for SAMHD1 negative cases; *p* = 0.016) (Fig. 2B). In the univariate analysis for PFS and OS, SAMHD1-negative cases were associated with longer TTP (*p* = 0.003) and prolonged OS (*p* = 0.014), like other well-characterized prognostic factors as are negative nodal status post NACT, pathological stage (I and II versus III) or adjuvant chemotherapy and adjuvant hormonal therapy among others (Table 2).

**Table 2:** Hazard ratios (HRs) and 95% confidence interval (CIs) for progression and death (univariate Cox regression models)

		Time to Progression		Overall Survival	
	N	HR (95% CI)	P		P
SAMHD1					
Negative < 1%	70	1	**0.003**	1	**0.014**
Positive ≥ 1%	31	2.74 (1.42–5.27)		2.15 (1.16–3.99)	
Not evaluable	50				
Post-NACT Nodal status					
Positive Negative	111	3.01 (1.53–5.93)	**0.001**	2.75 (1.22–6.22)	**0.014**
	40	1		1	
Post-NACT Ki67					
≥ 10 < 10 Not evaluable	26	1.69 (.85–3.34)	0.128	1.57 (0.79–3.12)	0.18
	72	1		1	
	53				
Pathological State					
I-II III	81	1	**0.001**	1	**0.015**
	70	2.73 (1.53–4.87)		1.70 (1.10–2.61)	
Estrogen Receptor					
Negative < 1% Positive ≥ 1%	59	0.69 (0.38–1.25)	0.22	0.37(0.20–0.69)	**0.002**
	92	1		1	
Progesterone Receptor					
Negative < 1% Positive ≥ 1%	85	0.72 (0.62–1.96)	0.72	0.52(0.27–0.99)	**0.047**
	66	1		1	
HER2 by FISH					
Negative Positive	120	1	0.47	1	0.13
	31	1.28 (0.65–2.47)		1.73(0.84–3.56)	
Post-NACT Histological grade					
I, II	75	1		1	
III Not evaluable	39	1.55(0.84–2.84)	0.15	2.55(1.37–4.76)	**0.003**
	37				
Subtypes					
Luminal A* Others	41	1	0.12	1	**0.009**
	110	1.73(0.85–3.50)		1.85(1.16–2.94)	
Menopausal status					
Premenopausal Postmenopausal	84	1.30 (0.73–2.31)	0.30	1.84(0.98–3.45)	0.057
	67	1		1	
Adjuvant Chemotherapy					
Yes Not	90 61	1 2.34 (1.21–4.52)	**0.01**	1 7.01(2.17–22.93)	**0.001**
Adjuvant RDT					
Yes Not	138	1	**0.002**	1	**0.04**
	13	1.02(0.31–3.31)		2.94(1.04–8.33)	
Adjuvant HT					
Yes Not	89	1	0.46	1	**0.015**
	62	0.80(0.44–1.44)		0.46(0.24–0.86)	

^*^5 years TTP, Luminal A greater than others HR (95%CI) 3.38 (1.19–9.57) p = 0.022

In bold, statistically significant p-values

More importantly, multivariate analysis for TTP showed also that SAMHD1-negative status and pathological stage (I and II versus III) were associated with prolonged TTP after NACT with anthracyclines-based regimens (*p* = 0.001 and *p* = 0.03, respectively) (Fig. 2C). Similar data were obtained on multivariate analysis for OS, where SAMHD1-negative status and negative post chemotherapy nodal status were the only variables associated with prolonged OS after NACT with anthracyclines-based regimens (*p* = 0.005 and *p* = 0.041, respectively) (Fig. 2D). Overall, our clinical data suggest that SAMHD1 expression is a relevant prognostic factor in breast cancer in the neoadjuvant setting.

### SAMHD1-KO breast cancer spheroids downregulate metabolic and immune signaling pathways

**Fig. 3:**
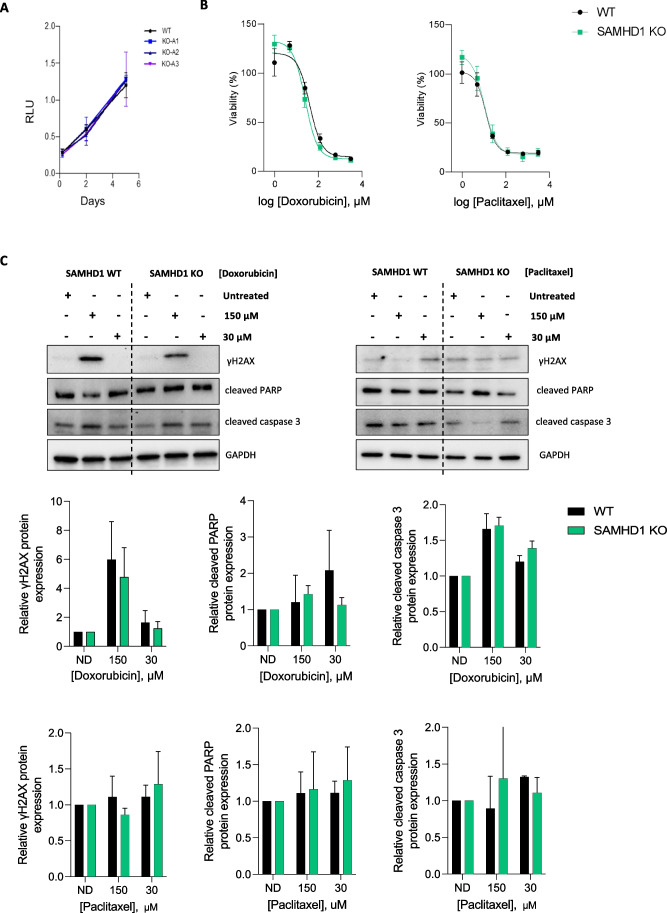
SAMHD1 depletion does not induce DNA damage after treatment with anthracyclines. **A** Growth curves of wild type (WT, black) and three different clones of SAMHD1 knockout (KO, blue) T47D cells. SAMHD1-KO did not show any difference in growth rate compared to WT when cultured in monolayer. Cell growth was measured using CellTiter-96® Aqueous One Solution Reagent and expressed as relative light units (RLU). Values represent mean ± SD from three different experiments. All measurements were performed in triplicates. **B** Dose–response curves showing cell viability of WT (black) and SAMHD1-knockout (green) T47D cells after 4 days of treatment with doxorubicin and paclitaxel. Values represent mean ± SD from three different experiments. **C** Representative western blots (upper panels) and quantification (bottom panels) showing γH2AX, cleaved PARP and cleaved caspase 3 expression in WT and SAMHD1-KO T47D cells treated with doxorubicin and paclitaxel for 24 h, relative to the corresponding untreated condition. Mean ± SEM of three different experiments is shown

To explore the functional mechanism underlying SAMHD1 impact on BC prognosis, we used our previously described T47D SAMHD1 *knock-out* (KO) cell model [30]to investigate putative effects of SAMHD1 expression in the response to neoadjuvant treatment, i.e., anthracyclines and taxanes. First, as SAMHD1 has been shown to significantly affect antiviral and cytotoxic efficacy of several anticancer treatments [38], cell proliferation capacity was measured in SAMHD1 KO cells treated with drugs used in the clinical cohort, i.e., doxorubicin, and paclitaxel. No differences were observed in the proliferation capacity of SAMHD1-KO cells (Fig. 3A, B), suggesting that SAMHD1 expression is not directly affecting cell proliferation capacity irrespective of the treatment. Then, we evaluated DNA damage induction and survival in wild-type and KO SAMHD1 treated cells by assessing γH2AX expression -a well-known marker for DNA double stranded breaks (DSBs), which has been widely used as a sensitive and reliable method for quantification of the DNA damage response. SAMHD1 KO cells did not show higher levels of γH2AX than wild type cells upon doxorubicin or paclitaxel treatment. Similar results were obtained when apoptosis levels were measured by evaluating cleaved caspase 3 and cleaved PARP protein expression by western blot (Fig. 3C). Thus, contrary to the reported effect upon platinum-derived drugs exposure [24, 39, 40], no significant differences were observed between SAMHD1 wild-type or KO cells treated with doxorubicin or paclitaxel (Fig. 3).

**Fig. 4:**
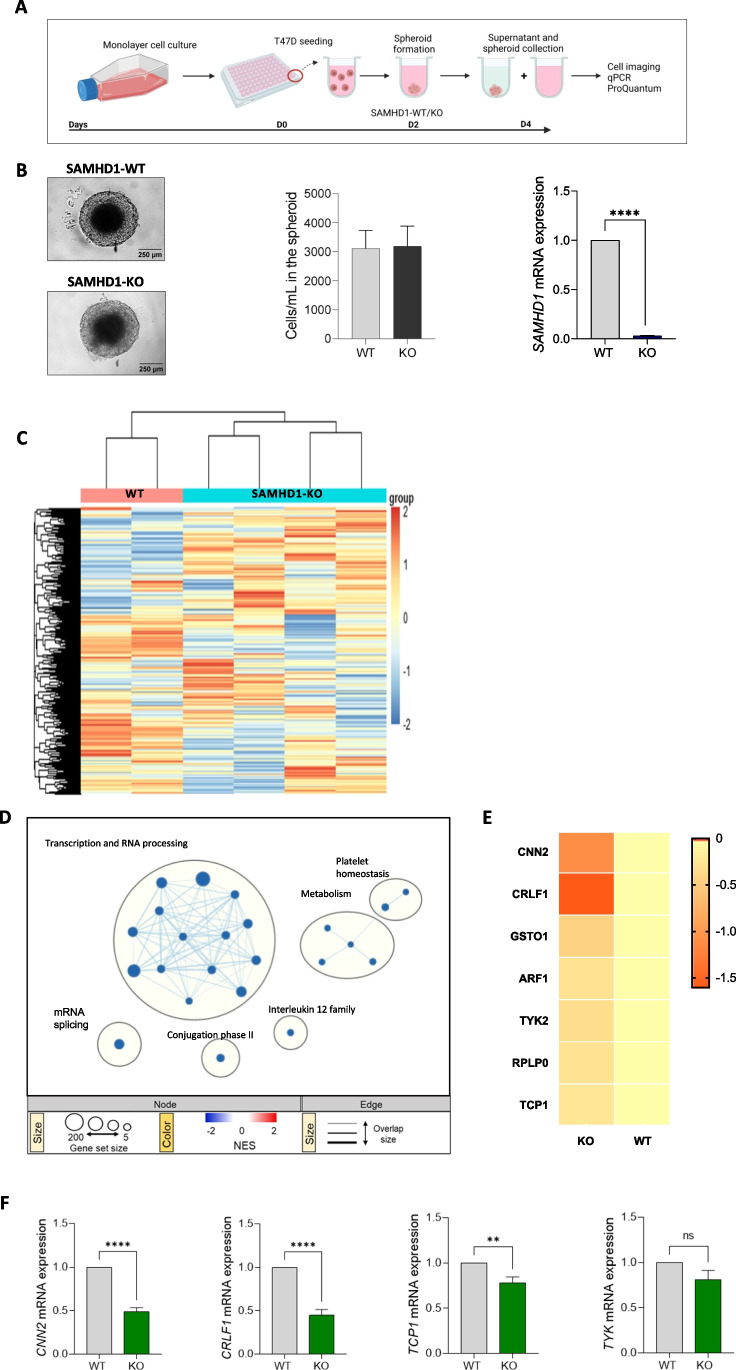
Gene expression changes in SAMHD1-KO and SAMHD1-WT T47D spheroids. **A** Workflow of wild-type (WT) and SAMHD1-knockout (KO) T47D spheroid generation. Spheroids were generated by seeding T47D cells on low adherent plates. On day 2, spheroids were already formed. Created with BioRender. **B** Brightfield microscope images of SAMHD1 wild-type (WT) (top) and SAMHD1-knockout (KO) (bottom) T47D spheroids showing no differences in spheroid structure or integrity. Images were taken 4 days after cells were seeding. Middle graph shows total cell count of WT (grey) and SAMHD1-KO (black) T47D spheroids. Cells were counted after spheroid disaggregation at day 4. All measurements were performed in triplicates. Right graph shows the lack of SAMHD1 mRNA expression in SAMHD1-KO T47D spheroids. All measurements were performed in triplicates. **C** Heatmap representation of gene expression changes in SAMHD1-KO and SAMHD1-WT T47D spheroids. Heatmap was generated by unsupervised hierarchical clustering of significantly differentially expressed genes (*p* < 0.05). **D** Reactome gene set enrichment map of significantly enriched pathways for SAMHD1-KO T47D spheroids. Reactome Gene set clusters are annotated, and nodes manually laid out for clarity. Single gene sets are highlighted and annotated in-text, underneath the cytoscape enrichment map. Node size represents number of genes, node color represents significance (NES), and edge thickness represents number of shared genes. **E** Gene expression of leading-edge genes with significant DEG from the Reactome IL-12 family gene set (gene set size *n* = 57). Values are relativized to SAMHD-WT T47D spheroids values, used as the reference set for leading-edge genes selection and ranking. Significantly down-regulated gene sets and genes are highlighted in orange. **F** mRNA expression of *CNN2*, *CRLF1*, *TCP1* and *TYK2* genes in T47D spheroids showing decreased expression in SAMHD1-depleted spheroids compared to SAMHD1-WT T47D spheroids (*p* = 0.0001, 0.0001, 0.0057 and 0.0944, respectively). Data represents mean ± SD of three additional spheroid experiments

Proliferation assays of monolayer cancer cells fail to model three-dimensional (3D) solid tumors, whereas cells cultured in 3D aggregates adopt the proper shape and experience cell–cell contacts and nutrient diffusion in all directions, representing more accurately the natural microenvironment of tumors and tissues. Thus, we set up a 3D cell culture model based on T47D spheroids, with the aim to better understand the functional consequences of SAMHD1 depletion. Wild-type and SAMHD1-KO T47D spheroids showed a tight spherical shape and stable appearance with an average size of 645 ± 30 μm and no significant differences were observed between wild-type vs. SAMHD1-KO T47D spheroids (Fig. 4A and B). Similarly, both wild-type and SAMHD1-KO T47D spheroids presented the typical oxygen-gradient structure with a dense cell core that may correspond with the necrotic zone as described before [41].

To further explore the mechanisms underlying SAMHD1 role, whole transcriptome profiling was performed on wild-type and SAMHD1-KO T47D spheroids. Hierarchical clustering of wild-type and SAMHD1-KO T47D spheroids using the union of all differentially expressed genes (DEGs) revealed distinct genetic signatures among them, while KO-spheroids derived from different clones presented more similar signatures, as expected (Fig. 4C and supplementary Fig. 1). To identify pathways specifically affected by the downregulation of SAMHD1 in T47D spheroids, we performed gene-set enrichment analysis (GSEA) using the Reactome gene-sets (Fig. 4D). Overall, depletion of SAMHD1 in T47D spheroids induced a global downregulation of several signaling pathways, especially at transcription and RNA processing level. More interestingly, downregulation of the IL-12 family was also observed in SAMHD1-KO T47D spheroids, suggesting that downregulation of SAMHD1 may influence immune signaling and response in tumors, in accordance with previous data demonstrating a link between SAMHD1 and IL-12 [42].

### Modeling of SAMHD1 knockout tumors exhibits reduced interleukin signaling

To characterize the putative effect of differential interleukin signaling of SAMHD1-KO T47D spheroids, we conducted a more detailed evaluation of the IL-12 downregulated pathway. Examination of the leading-edge genes of the IL-12 family, indicated that there were 7 genes that contributed the most (Fig. 4E). A group of four genes, including *CNN2* (Calponin), *CRLF1* (Cytokine receptor like factor 1), *TYK2* (Tyrosine kinase 2) and *TCP1* (T-complex protein 1 subunit alpha) with reported expression in breast tissues were selected for further study (based on Human Protein Atlas proteinatlas.org), after confirming their downregulation upon SAMHD1 depletion in additional spheroid samples by quantitative PCR analysis (Fig. 4F).

**Fig. 5:**
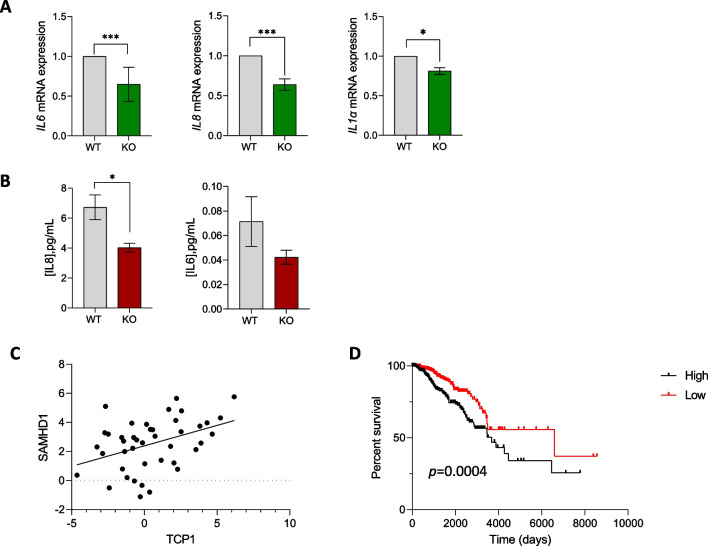
SAMHD1-KO T47D spheroids presented decreased pro-inflammatory cytokine expression compared to SAMHD1-WT spheroids. **A** Gene expression of distinct proinflammatory cytokines (IL6, IL8, IL1α) in breast tumor spheroids. IL6, IL8, IL1α were significantly decreased in SAMHD1-KO spheroids (*p* = .001, < .0001 and .0475, respectively). Data represents mean ± SD of three additional spheroid experiments. **B** Evaluation of protein expression in supernatants from spheroids cultured for 4 days. SAMHD1-KO T47D spheroids present decreased IL8 and IL6 (*p* = .0277 for IL8), further confirming the RNA expression results. Measurements were performed in triplicate in three additional spheroid samples. **C** Correlation between *SAMHD1* and *TCP1* expression levels in RNA extracted from tumor biopsies. **D** Kaplan–Meier curves of overall survival in breast cancer patients from the Human Protein Atlas (HPA) datasets for *TCP1* gene divided by high (black line) or low (red line) expression level. According to HPA, *TCP1* is a prognostic factor in breast cancer (*p* = 0.0004). Cut off for determining high or low expression level was calculated using medium FPKM (FPKM = 36.85). Data from men and stages I and IV was excluded for a better representation of the cohort described in this paper

These differentially expressed genes are mostly involved in the regulation of immune-mediated signaling, thus, we further evaluated the expression of other interleukins in SAMHD1-KO T47D spheroids. Interestingly, reduced production of IL6, IL8 and IL1α were also observed, both at mRNA level (Fig. 5A, Supplementary Fig. 1B) and protein level, measured as IL6 and IL8 production in T47D spheroid culture supernatant (Fig. 5B). In addition, transient downregulation of SAMHD1 by RNAi in MCF7 cell line showed no differences in MCF7 spheroid integrity or proliferation and a reduction of *IL6* and *IL8* expression (Supplementary Fig. 2). This was similar to what had been previously observed in T47D spheroids, further confirming the specificity of the effect.

To validate the involvement of the IL-12 family pathway *in vivo*, we selected a group of patients (*n* = 47) with available biopsies from primary tumors to quantify gene expression of key genes, i.e., *SAMHD1* and IL-12 family leading-edge genes. mRNA expression of *SAMHD1*, *CNN2, CRLF1* and *TCP1* genes was measured by qRT-PCR in 42 patients. Correlation analysis was performed in samples with detectable expression for all genes tested, except *CRLF1,* whose expression was undetectable in most samples and thus, we did not explore further its putative role. No significant correlation was observed between *SAMHD1* and *CNN2* gene (data not shown). However, a positive correlation between *SAMHD1* and *TCP1* expression was identified (*p* = 0.0081), confirming the transcriptomic data and pointing towards TCP1 as an additional factor determining SAMHD1 effect in breast cancer (Fig. 4C). Interestingly, TCP1 is part of chaperonin multi-subunit protein-folding complex (TRiC or CCT) that has been previously described to interact with many oncoproteins and mutant tumor suppressors. To this end, using TCGA data on survival probability in BC, we confirmed that *TCP1* low expressing tumors had better prognosis, similar to the data obtained with SAMHD1 expression in our cohort, while *CNN2*, *TYK2* and *CRLF1* expression do not affect survival, in agreement with our data from RNA expression in tumor biopsies (Fig. 4D and Supplementary Fig. 3).

### SAMHD1-KO T47D spheroids are less susceptible to myeloid and neutrophil infiltration

**Fig. 6:**
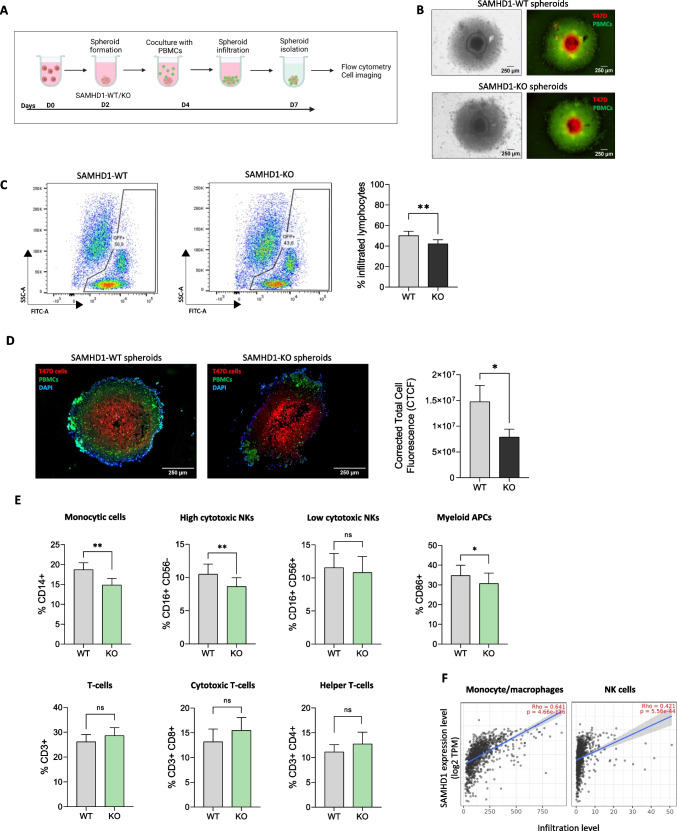
SAMHD1-KO T47D spheroids presented decreased immune infiltration than SAMHD1-WT spheroids. **A** Workflow of coculture generation of T47D spheroids and primary PBMCs. At day 4, when spheroids were complete formed, cocultures were established by adding peripheral blood mononuclear cells (PBMCs) from healthy donors. After three days, infiltration into the T47D spheroids was evaluated by multipanel flow cytometry. Imaging of spheroid composition and cell invasion was performed by microscopy. Created with BioRender. **B** Representative brightfield (left) and fluorescence microscopy (right) images from cocultures of SAMHD1-WT (top) and SAMHD1-KO (bottom) T47D spheroids and PBMCs 3 days after cocultures were established. T47D cells were pre-stained with CellTracker™ Red CMPTX Dye. PBMCs were stained with CellTracker™ Green CMFDA Dye. **C** Representative cytometry plots (left) and quantification (right) of immune cells infiltrated into the spheroids. SAMHD1-KO T47D spheroids presented decreased % of infiltrated lymphocytes (*p* = .0014) compared to SAMHD1-WT. All measurements were performed at least in triplicates. **D** Representative fluorescence images (left) and CTCF quantification (right) of sections of paraffin-embedded PBMCs and T47D spheroid cocultures. Quantification of corrected total cell fluorescence (CTCF) showing decreased infiltration into the SAMHD1-KO spheroids (*p* = . 0484). SAMHD1-KO T47D spheroids presented decreased immune infiltration than SAMHD1-WT. **E** Immunophenotypic characterization of infiltrated cells into the T47D spheroids. Infiltrated immune cells into spheroids were evaluated by multipanel flow cytometry. SAMHD1-KO spheroids presented significant decreased of myeloid APCs (CD86 +), monocytic cells (CD14 +) and high cytotoxic NKs (CD16 + CD56 +) (*p* = .0156, .0053 and .0025, respectively), whereas helper T cells (CD3 + CD4 +), cytotoxic T cells (CD3 + CD8 +) and low cytotoxic NKs (CD16 + CD56-) did not differ. All measurements were performed at least in triplicates. (F) Correlation of SAMHD1 expression levels with monocyte/macrophage and NK cell infiltration in BC, as implemented in the TIMER resource

Accumulating evidence have shown that the interaction between cancer cells and the TME, specifically the immune microenvironment, is a vital factor in tumor progression and therapy [43]. Thus, as interleukins and their signaling pathways identified above, are potent chemoattractant for immune cells and may determine tumor immune infiltration, we evaluated the capacity of immune cells to infiltrate in SAMHD1 KO and WT T47D spheroids. We developed an *in vitro* coculture system including T47D spheroids and peripheral blood mononuclear cells (PBMCs) derived from healthy donors (Fig. 6A). No significant changes in T47D spheroid integrity were observed between WT and KO-SAMHD1 cocultures (Fig. 6B). Then, the number of immune cells infiltrated into the tumor spheroids was quantified by flow cytometry and immunofluorescence. Interestingly, a significant reduction in the number of infiltrating PBMCs was observed in SAMHD1-KO compared to WT T47D spheroids, presumably due to of the reduced IL-mediated signaling upon *SAMHD1* depletion (Fig. 6C and D). Next, the type of infiltrated PBMCs was evaluated by immunophenotypic characterization through flow cytometry (Supplementary Fig. 4), identifying major changes in myeloid antigen presenting cells, monocytic cells and high cytotoxic NK cells (*p* = 0.0156, 0.0053 and 0.0025, respectively), whereas CD4 + and CD8 + T lymphocytes did not differ (Fig. 6E). Overall, our data suggest that impaired IL-mediated signaling observed in SAMHD1-KO T47D spheroids have an impact on preferentially myeloid and NK cell subsets. Hence, we determined whether SAMHD1 expression correlated with the immune infiltration level in BC patients by calculating the coefficient of SAMHD1 expression and the distinct immune cell types evaluated experimentally in TIMER [43]. SAMHD1 expression positively correlated with monocytes cells and NK cells, i.e., the lower SAMHD1 expression the lesser infiltrated cells. These results are in agreement with the data described above in the *in vitro* models (Fig. 6E) and confirm the role of the SAMHD1 as a regulator of BC prognosis through the induction of changes in immune response and TME.

## Discussion

NACT is a valuable clinical and research tool with great potential for advancing in BC treatments. Albeit multiple studies have shown that achieving a pCR is associated with improved DFS and OS, the lack of validated surrogate biomarkers in patients not achieving a pCR remains a major challenge. Patients who do not achieve a pCR post-NACT constitute an unmet need in BC but also represent an opportunity to identify new markers or test novel adjuvant treatments enabling personalized therapy. Here, based on previous data pointing towards a prognostic role of SAMHD1 in BC [30], we describe the use of SAMHD1 expression in tumor biopsies post-surgery as a novel prognostic biomarker in early BC patients with residual disease after NACT, where there is still the need to develop effective biomarkers and more efficient alternative treatment options.

In recent years, SAMHD1 has been proposed as a prognostic marker mainly in different types of hematological cancers, but also to a lesser extent in different solid tumors such as NSCLC and colorectal cancer [44]. Recently, we reported the value of SAMHD1 in several solid tumors, including the first evidence of its negative prognostic value in advanced BC [30]. Here, in line with our previous data, SAMHD1 expression was also identified as a biomarker determining prognosis in early cases of BC who did not achieve pCR after NACT, being SAMHD1 expression linked to shorter TTP and OS. Accordingly, SAMHD1 expression was significantly associated with tumor grade, being poorly differentiated, high-grade tumors those with the highest proportion of SAMHD1 positive cases and showing significantly higher levels of the cell proliferation marker Ki67. These data point towards a relevant role for SAMHD1 in cell proliferation and BC development as previously suggested [44], albeit contrarywise to existing evidences derived mainly from the study of hematological cancers where it was assumed that SAMHD1 depletion led to an increase in dNTP availability, thereby favoring cell proliferation [39, 40]. Noteworthy, our data are in consonance with previously published data from The Cancer Genome Atlas database, where high SAMHD1 expression was associated with reduced OS in adult white cancer patients [45]. Moreover and despite that the clinical impact of SAMHD1 in solid tumors has been evaluated in a limited number of studies, data from other cancer types also support the negative prognosis here described for SAMHD1-expressing tumors, including (i) our previous study on ovarian and lung cohorts [30], (ii) colorectal cancer where high SAMHD1 expression levels in tumors correlated to increased risk of metastasis formation [46], (iii) untreated classical Hodgkin lymphoma, where SAMHD1 was an independent adverse prognostic factor [47], (iv) NSCLC EGFR mutated cancer patients where SAMHD1 serum levels were significantly increased when compared with normal controls upon cancer progression [48] and (v) in mantle cell lymphoma patients treated with chemotherapy [49].

From a functional point of view, the cellular functions of SAMHD1 are far beyond the regulation of intracellular dNTP pool, where reduced dNTPase activity of SAMHD1 might lead to persistently high levels of dNTPs and abnormal cell proliferation [50–52]. Several studies have reported that SAMHD1 interacts with cyclin/CDK complexes [53], USP18 and S-phase kinase-associated protein 2 [54, 55], which are involved in the regulation of cell proliferation [53, 55] and SAMHD1 is also recruited to DNA repair foci in response to DNA damage [56]. Additional pieces of evidence also demonstrate the role of SAMHD1 in innate immune sensing, having important implications in cytokine production. Finally, several studies have shown that SAMHD1, due to its dNTPase activity, can also have a significant impact on the efficacy of nucleoside-based chemotherapies [57]. Therefore, SAMHD1 can be placed at the crossroads of various cellular processes that may influence cancer cell biology via multiple dNTPase-dependent or -independent mechanisms, but the underlying mechanisms of SAMHD1 role in the induction and regulation of tumorigenesis remain unknown. Indeed, both clinical and preclinical evidence here shown suggest that SAMHD1 does not affect doxorubicin or paclitaxel-based treatment, as no changes in treatment efficacy were observed in patients but also drug efficacy was similar in wild-type or KO cells, as well as no changes in expression of DNA damage and/or apoptotic markers were reported.

Thus, to explore the mechanisms underlying SAMHD1 effect on BC prognosis, whole transcriptome profiling was performed on wild-type and SAMHD1-KO T47D spheroids. Interestingly, gene set enrichment analysis identified as one of the most significant pathways a downregulation of the IL-12-family signaling. Indeed, IL-12 signaling has been previously linked to the regulation of cell polarization through the regulation of SAMHD1 in HIV-1 infection [58], showing also a direct correlation between SAMHD1 expression and IL-12/IL-18 presence. In our transcriptomics analysis, IL12-family signaling was mainly mediated by a series of genes involved in the regulation of the innate immune system and transcription of associated genes but not the IL-12 cytokine itself, including *CNN2*, whose depletion restricts proinflammatory activation [59]; *CRLF1*, involved in regulating the immune system during inflammatory response; *TYK2*, a member of the Janus kinases (JAKs) family, that through its association with cytokine receptors promulgates cytokine signals by phosphorylating receptor subunits and *TCP1*, a molecular chaperone that is a member of the chaperonin containing TCP1 complex (CCT or TRiC) [60]. Although their downregulation was confirmed in additional *in vitro* experiments, only *TCP1* could be confirmed in patients, i.e. TCP1-low-expressing tumors showed also low-levels of SAMHD1 expression by qRT-PCR on tumor biopsies. Similarly, low TCP1 expression was associated with increased survival using Human Protein Atlas (HPA) data on BC, showing the same trend as in our series here reported for SAMHD1 expression. Different studies have previously linked expression levels of different CCT subunits in various cancers, such as CCT2 in prostate, breast and lung cancers [61–63] and CCT3 in hepatocellular carcinoma (HCC) [64]. Specifically in BC, increased expression of the CCT complex has been linked to enhanced *in vitro* growth/survival [65] and worse prognosis in patients [66] and specifically, elevated expression of *TCP1* was associated with poor clinical outcomes in BC. Although molecular mechanisms underlying TCP1 role in promoting cancer are unknown, our data point towards SAMHD1 as a key factor, confirming the prognostic value of SAMHD1 through the modulation of TCP1 levels, which may putatively also affect IL signaling.

In this sense, we assessed whether SAMHD1 depletion in T47D spheroids also impairs the expression of other pro-inflammatory ILs, confirming a downregulation of IL-6 and IL-8 *in vitro* in SAMHD1-KO T47D spheroids. Interestingly, basal low levels of IL-6 and IL-8 correlate with improved OS in metastatic BC [67] and other trials have reported the individual prognostic role of IL-6 [68, 69] and IL8 [70] in BC. Overall, our data point towards the idea that SAMHD1 may play a role as a surrogate biomarker of BC prognosis, defined by its capacity to modulate innate immune response, through a process in which the chaperone complex TCP1 plays a key role. Indeed, the role of cytokines, including IL-6 and IL-8 as potent chemoattractants for different immune cells is well described [76]. Moreover, expression of CCT subunits has also been related to decreased immune infiltrates in breast and colorectal cancer in *in sillico* analysis. Thus, we hypothesized that the above-described functional consequences of SAMHD1 depletion in breast cancer spheroids *in vitro* may also affect immune infiltration capacity, i.e., by limiting either the number or type of immune cells in tumor spheroids. Interestingly, in our heterotypic model of T47D spheroids and primary PBMCs, we found that SAMHD1-KO condition was associated with less infiltration of immune cells, as should be expected by the reduced expression of ILs in T47D spheroids. In addition, a more detailed immunophenotypic characterization of the subtype of infiltrating cells, identified cells from the monocytic lineage and NK cells as those subtypes to which SAMHD1 depletion exerted a more significant effect, whereas CD4 + and CD8 + T lymphocytes did not differ. Despite the robustness of our observations, an important limitation of our experimental setting is the inability to model the gradient of IL concentration generated between the outer and inner areas of the spheroid upon SAMHD1 depletion. Thus, we sought to further validate our in tissues from BC patients using the TIMER resource, overall showing similar data than those previously reported for distinct CCT subunits in BC and colorectal cancer. These results further suggest that SAMHD1 depletion impacts tumor infiltration capacity, through its ability to deregulate innate immune signaling.

Taken together, our results indicate that SAMHD1 expression exerts a pro-tumorigenic effect in breast cancer, through a process that involves the interaction between tumors cells and TME putatively through the differential regulation of inflammatory intratumoral response. Several promising strategies are currently being evaluated in the post-neoadjuvant setting, such as immunotherapy and targeted therapies, whose success is linked to a more precise knowledge of the tumor molecular and genomic characteristics together with the characterization of the immune infiltration landscape. Based on both the clinical importance of SAMHD1 and the detailed knowledge of its functions and regulation mechanisms, SAMHD1 may become an interesting target for cancer treatment.

## Supplementary Information

Below is the link to the electronic supplementary material.

Supplementary file1 (PPTX 3105 KB) **Supplementary figure 1.** (A) **Bar plots of top 30 differentially enriched genes (DEG) for SAMHD1-KO relative to SAMHD1-WT T47D spheroids**, based on Log2 gene expression (Log2FC) and *p*<0.05. Significantly down- or up-regulated DEG are highlighted in blue or red, respectively. (B) Normalized counts of *IL8* and *IL1A* genes from RNAseq data in T47D WT spheroids (grey bars) or SAMHD1-KO spheroids (blue bars), confirming expression downregulation upon SAMHD1 depletion, although it did not reach statistical significance. **Supplementary figure 2. SAMHD1-depleted MCF7 spheroids show a decrease in IL6 expression but not differences in integrity or spheroid structure. **(A) Brightfield microscope images of SAMHD1-WT (left) and SAMHD1-depleted (right) MCF7 spheroids showing no differences in spheroid structure or integrity. Images were taken 4 days after cells were seeding. Right graph shows the total cell count of WT (grey) and SAMHD1-KO (black) MCF7 spheroids. Cells were counted after MCF7spheroid disaggregation at day 4. All measurements were performed in triplicates. (B) SAMHD1 (left) and IL6 (right) mRNA showing specific siRNA-mediated inhibition of SAMHD1 and the subsequent decrease in IL6 in MCF-7 spheroids (*p*=0.0036 and 0.0003 respectively). **Supplementary figure 3. Kaplan-Meier curves of overall survival from the Human Protein Atlas datasets for *CNN2*, *TYK2* and *CRLF1* genes divided by high (black line) or low (red line) expression level in breast cancer patients. **According to HPA, any of these genes are a prognostic factor in breast cancer (log-rank test *p*=0.0215; 0.14, 0.779 respectively). Cut off for determining high or low expression level was calculated using medium FPKM that was 37.2; 9.62 and 1.01 respectively for each gene. Data from men and stages I and IV was excluded for a better representation of the cohort described in this paper. **Supplementary figure 4. Gating strategy for the immunophenotypic characterization of PBMCs by flow cytometry. **Different cell populations were defined based on the following expression combinations gated on the live singlet lymphocytes: monocytic cells (CD14+), high cytotoxic natural killer cells (NKs) (CD16+CD56-), low cytotoxic NKs (CD16+ CD56+), myeloid antigen presenting cells (APCs) (CD86+), T cells (CD3+), cytotoxic T cells (CD3+ CD8+) and helper T cells (CD3+ CD4+).

Supplementary file2 (DOCX 23 KB)

## Data Availability

Data generated during this study are included in this published article and its supplementary information files. Sequencing files can be accessed on gene expression omnibus repository (GSE224678). All other datasets used are available from the corresponding author on request.
