# Supplementary material for: SAMHD1 expression is a surrogate marker of immune infiltration and determines prognosis after neoadjuvant chemotherapy in early breast cancer
Source: Cell Oncol (Dordr). 2023 Sep 4;47(1):189–208. doi: 10.1007/s13402-023-00862-1 (PMC10899429; doi:10.1007/s13402-023-00862-1)
Supplement: Supplementary file 1 — Supplementary file1 (PPTX 3105 KB) Supplementary figure 1. (A) Bar plots of top 30 differentially enriched genes (DEG) for SAMHD1-KO relative to SAMHD1-WT T47D spheroids, based on Log2 gene expression (Log2FC) and p<0.05. Significantly down- or up-regulated DEG are highlighted in blue or red, respectively. (B) Normalized counts of IL8 and IL1A genes from RNAseq data in T47D WT spheroids (grey bars) or SAMHD1-KO spheroids (blue bars), confirming expression downregulation upon SAMHD1 depletion, although it did not reach statistical significance. Supplementary figure 2. SAMHD1-depleted MCF7 spheroids show a decrease in IL6 expression but not differences in integrity or spheroid structure. (A) Brightfield microscope images of SAMHD1-WT (left) and SAMHD1-depleted (right) MCF7 spheroids showing no differences in spheroid structure or integrity. Images were taken 4 days after cells were seeding. Right graph shows the total cell count of WT (grey) and SAMHD1-KO (black) MCF7 spheroids. Cells were counted after MCF7spheroid disaggregation at day 4. All measurements were performed in triplicates. (B) SAMHD1 (left) and IL6 (right) mRNA showing specific siRNA-mediated inhibition of SAMHD1 and the subsequent decrease in IL6 in MCF-7 spheroids (p=0.0036 and 0.0003 respectively). Supplementary figure 3. Kaplan-Meier curves of overall survival from the Human Protein Atlas datasets for CNN2, TYK2 and CRLF1 genes divided by high (black line) or low (red line) expression level in breast cancer patients. According to HPA, any of these genes are a prognostic factor in breast cancer (log-rank test p=0.0215; 0.14, 0.779 respectively). Cut off for determining high or low expression level was calculated using medium FPKM that was 37.2; 9.62 and 1.01 respectively for each gene. Data from men and stages I and IV was excluded for a better representation of the cohort described in this paper. Supplementary figure 4. Gating strategy for the immunophenotypic characterization of PBMCs by flow cytom [file 13402_2023_862_MOESM1_ESM.pptx]

## Slide 1
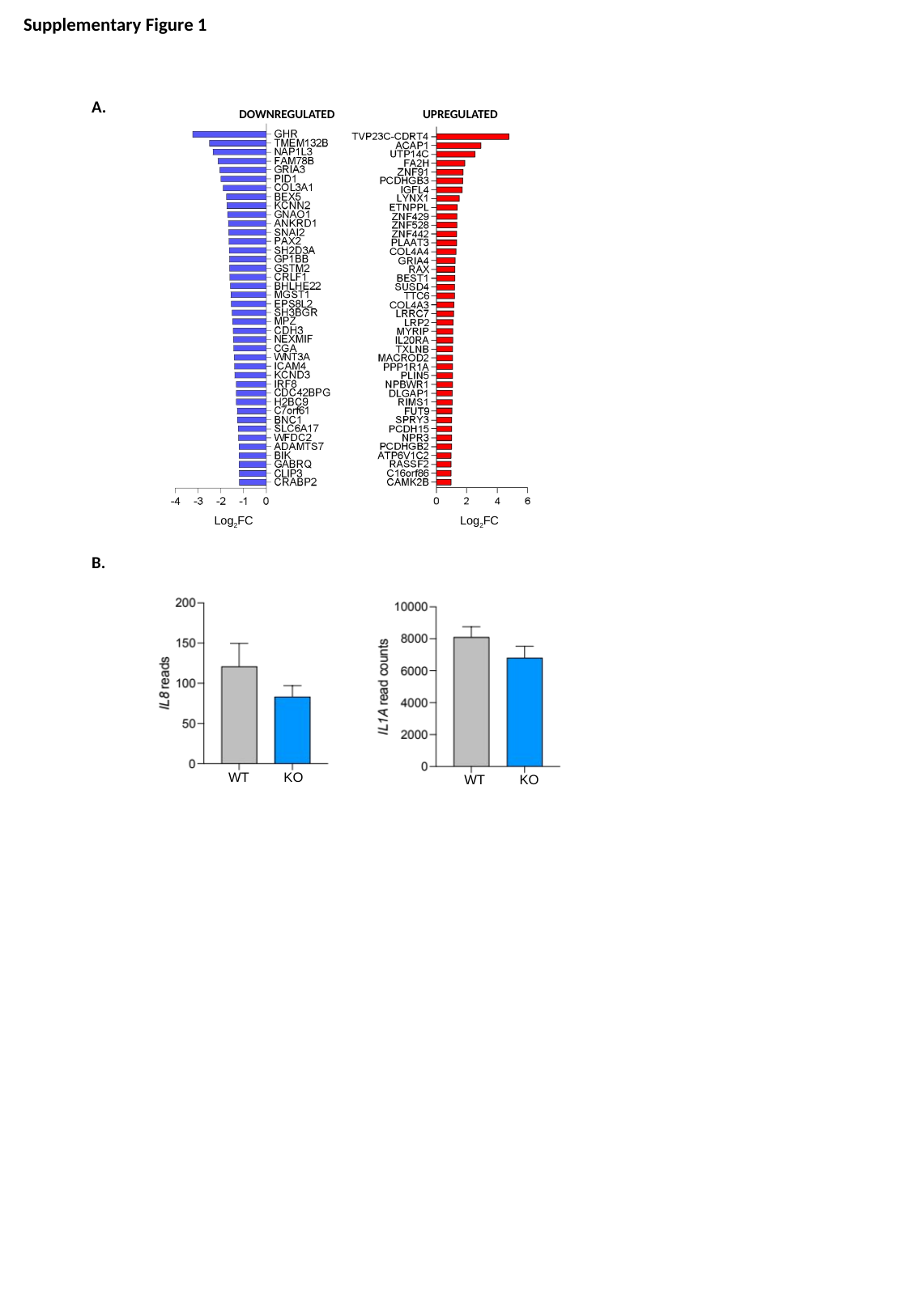

Supplementary Figure 1
A.
DOWNREGULATED
UPREGULATED
Log2FC
Log2FC
B.
WT KO
WT KO

## Slide 2
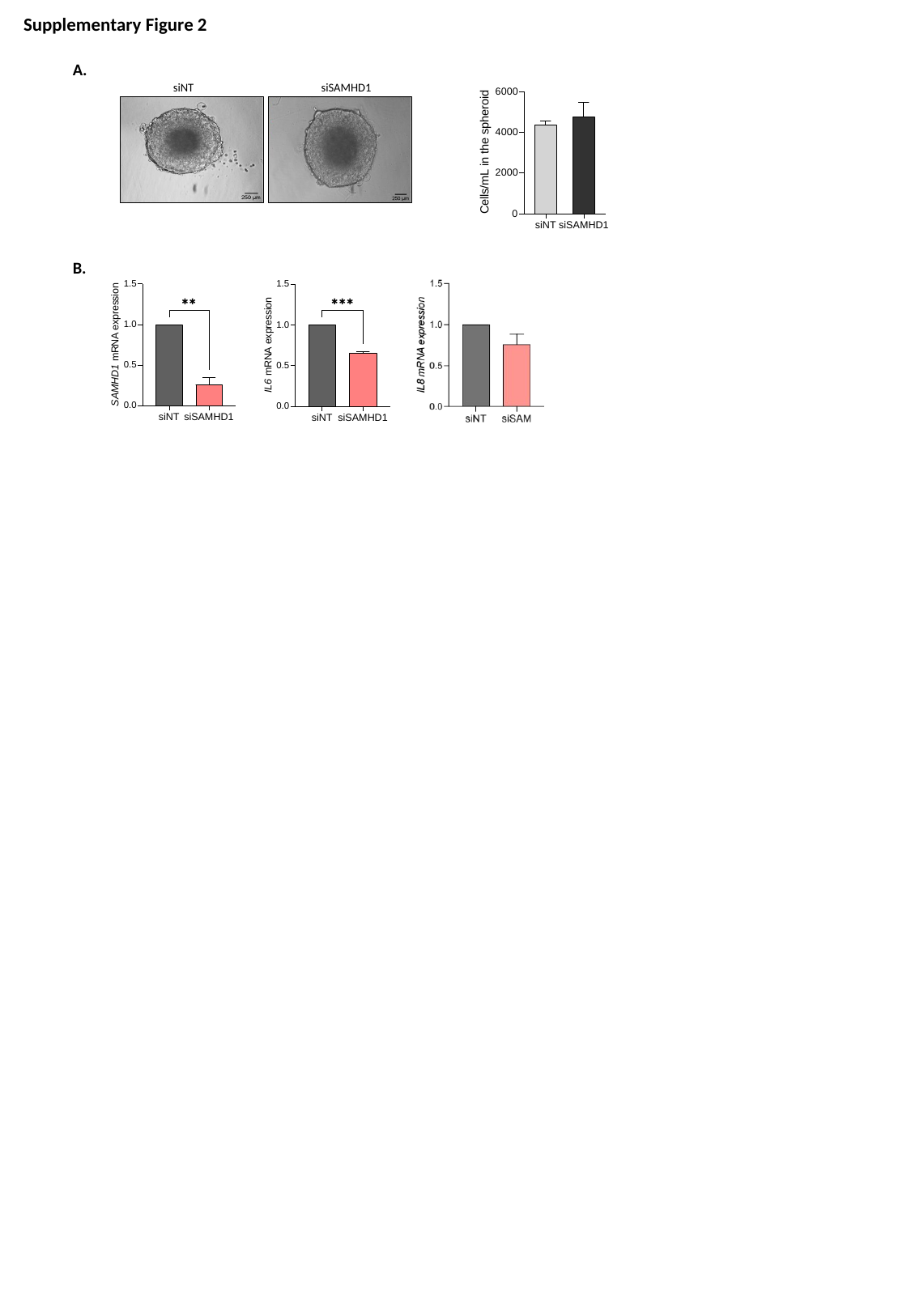

Supplementary Figure 2
A.
siNT
siSAMHD1
B.

## Slide 3
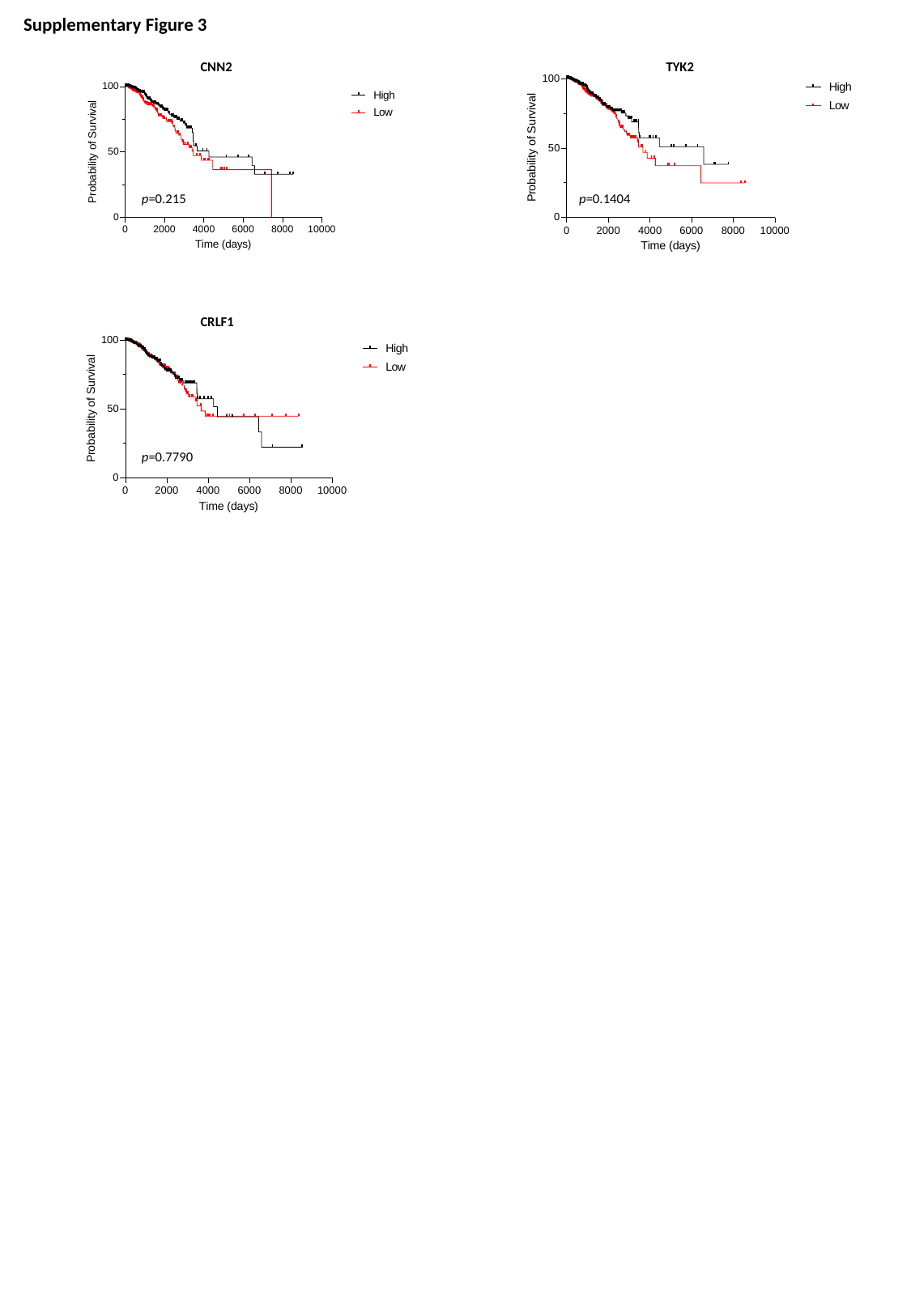

Supplementary Figure 3
CNN2
TYK2
p=0.215
p=0.1404
CRLF1
p=0.7790

## Slide 4
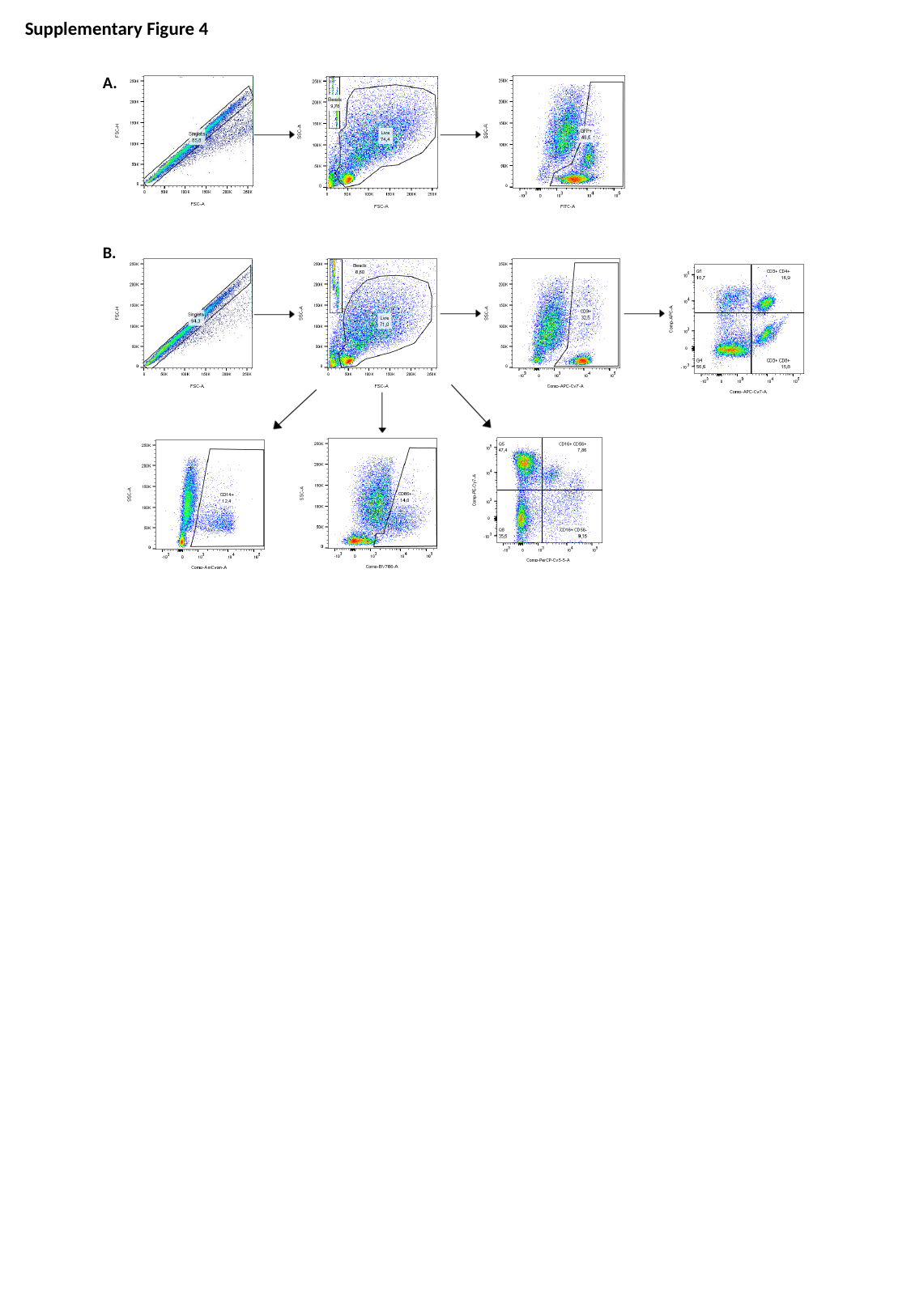

Supplementary Figure 4
A.
B.

## Slide 5
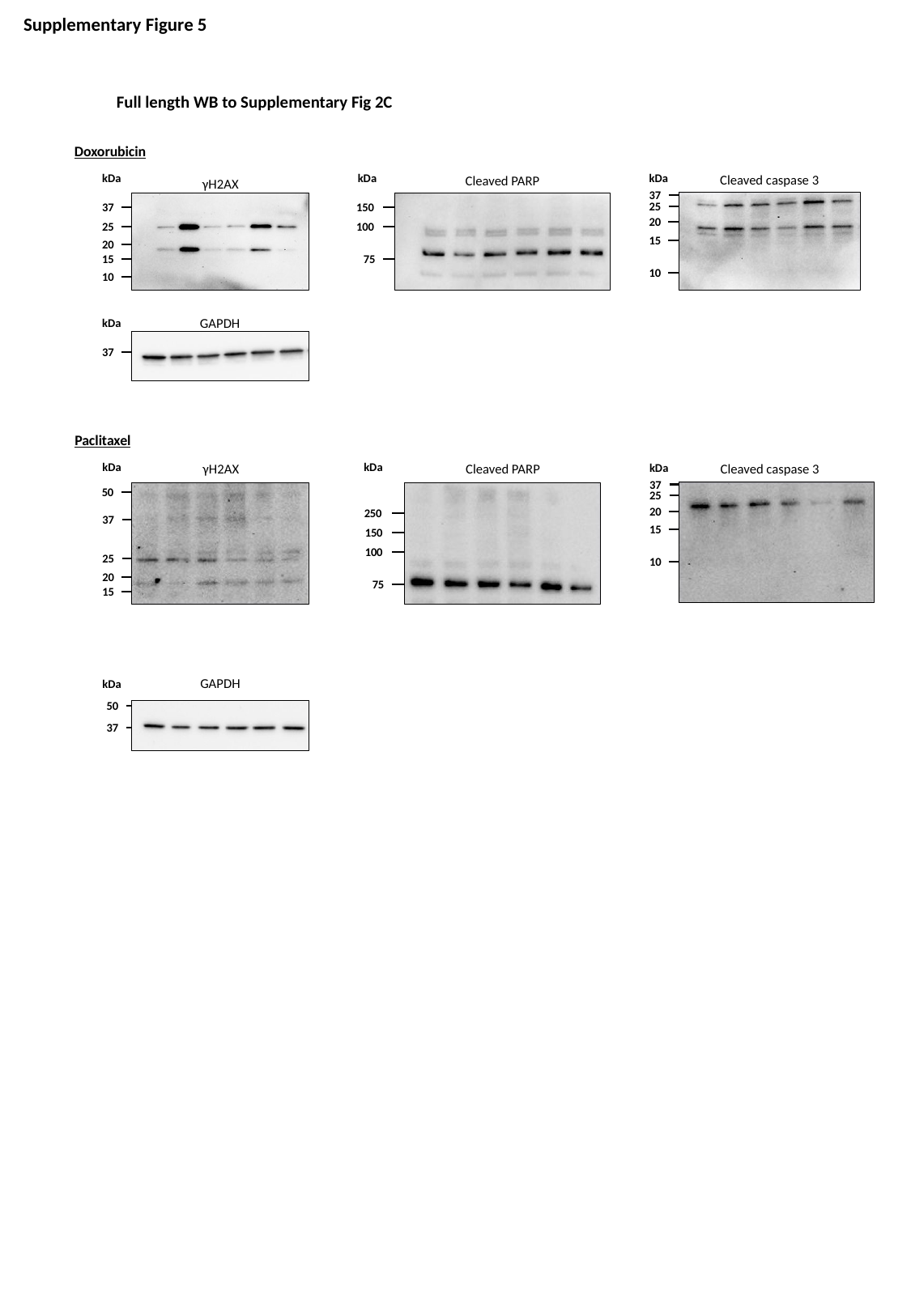

Supplementary Figure 5
Full length WB to Supplementary Fig 2C
Doxorubicin
kDa
kDa
kDa
Cleaved caspase 3
Cleaved PARP
γH2AX
37
25
37
150
20
25
100
15
20
15
75
10
10
GAPDH
kDa
37
Paclitaxel
kDa
kDa
γH2AX
Cleaved PARP
Cleaved caspase 3
kDa
37
50
25
20
250
37
15
150
100
25
10
20
75
15
GAPDH
kDa
50
37
