# Supplementary material for: SAMHD1 expression is a surrogate marker of immune infiltration and determines prognosis after neoadjuvant chemotherapy in early breast cancer
Source: Cell Oncol (Dordr). 2023 Sep 4;47(1):189–208. doi: 10.1007/s13402-023-00862-1 (PMC10899429; doi:10.1007/s13402-023-00862-1)
Supplement: Supplementary file 2 — Supplementary file2 (DOCX 23 KB) [file 13402_2023_862_MOESM2_ESM.docx]

**Supplementary Tables**

**Supplementary table 1. Clinical characteristics of NACT cohort of breast cancer patients.**

| **Variable** | **All patients (n=182)** | **Excluding pCR cases (N=151)** |  |
| --- | --- | --- | --- |
| **Age** (y), Mean (IQR), DT | 50.07 (25-79) | 50.28 (24-79) | .319 |
| **Menopausal status, n (%)**  Yes  Not | 78 (42.9%)  104 (57.1%) | 67 (55.6%)  84 (44.4%) | .240 |
| **Clinical Stage**  IIA  IIB  IIIA  IIIB  IIIC | 31 (17%)  43 (23.6%)  67 (36.8%)  38 (20.9%)  3 (1.7%) | 25 (16.6%)  34 (22.5%)  57 (37.7%)  33 (21.9%)  2 (1.3%) | .780 |
| **Clinical Stage**  II  III | 74 (40.7%)  108 (59.3%) | 59 (39.1%)  92 (60.9%) | .222 |
| **Nodal status (Dg)**  Negative  Positive | 45 (24.7%)  137 (75.3%) | 40 (26.5%)  111 (73.5%) | .161 |
| **Estrogen Receptor (ER)**  <1  1-10  >10 | 67 (36.8%)  12 (6.6%)  103 (56.6%) | 59 (39.1%)  11 (7.3%)  81 (53.6%) | .198 |
| **Progesterone Receptor (PR)**  <1  1-10  >10 | 99 (54.4%)  7 (3.8%)  76 (41.8%) | 85 (56.3%)  7 (4.6%)  59 (39.1%) | .169 |
| **HER2 (IHQ&FISH)**  Negative  Positive | 144 (79.1%)  38 (20.9%) | 120 (79.5%)  31 (20.5%) | .482 |
| **Ki67 Post surgery (mean)** | 12.43(0-80) | 12.67 (0-80) | .571 |
| **Histological grade post-surgery**  **I**  **II**  **III**  **Unknown** | 13 (7.1%)  67 (36.8%)  46 (25.3%)  56 (30.8%) | 13 (8.6%)  62 (41.1%)  39 (25.8%)  37 (24.5%) | .565 |
| ***Subtypes**  Luminal A  Luminal B  Luminal B HER2 positive  HER2 positive  Triple Negative | 44 (24.2%)  40 (21.9%)  18 (9.8%)  22 (12.2%)  58 (31.9%) | 41 (27.2%)  38 (25.2%)  14 (9.2%)  17 (11.2%)  41 (27.2%) | .663 |
| **Pre-surgery Chemotherapy**  Chemotherapy (Anthracyclines and taxanes) plus Trastuzumab  Anthracycline based regimens  Anthracycline and taxane based regimens | 16 (8.8%)  52 (28.6%)  114 (62.6%) | 12 (7.9%)  47 (31.1%)  92 (61%) | .206 |
| **Clinical response**  CR  PR  ED  PD | 57 (31.3%)  101 (55.5%)  20 (11%)  4 (2.2%) | 46 (30.5%)  83 (55.0%)  18 (11.9%)  4 (2.6%) | .621 |
| **Type of surgery**  Tumorectomy + AND  Mastectomy + AND  Tumorectomy | 67 (36.8%)  112 (61.5%)  3 (1.7%) | 57 (37.7%)  91 (60.3%)  3 (2%) | .589 |
| **Complementary Chemotherapy (including Trastuzumab)**  Yes  Not | 110 (60.4%)  72 (39.6%) | 90 (59.6%)  61 (40.4%) | .383 |
| **Complementary Hormonotherapy**  Yes  Not | 111 (61%)  71 (39%) | 89 (58.9%)  62 (41.1%) | .147 |
| **Complementary Radiotherapy**  Yes  Not | 167 (91.8%)  15 (8.2%) | 138 (91.4%)  13 (8.6%) | .512 |
| **Recurrence**  Yes  Not | 65 (35.7%)  117 (64.3%) | 51 (33.8%)  100 (66.2%) | .159 |
| **Type of relapse**  Only Local  Distant (with or without local) | 3 (4.6%)  62 (95.4%) | 2 (4%)  49 (96%) | .512 |

*Luminal A: ER positive,PR>20%, HER2 negative ki67<14%; Luminal B: ER positive, PR<20%, HER2 negative, and/or ki67≥14%; Luminal B HER2 positive: ER and/or PR positive, HER2 positive;  HER2: HER2 positive;Triple Negative: ER negative, PR negative, HER2 negative

**Supplementary table 2. Primer sequences used for the amplification of SAMHD1 coding region.**

| **Exon** | **bp** | **Sequence forward** | **Sequence reverse** |
| --- | --- | --- | --- |
| **1** | 460 | (a) GCCAATAGGCTGCCAATACT  (b) ATGCAGCGAGCCGATTC | (a) TGTCTTGTAGTCGGGATGGA  (b) GCTACCTCGGATGTTCTTCAG |
| **2** | 182 | TGGATCTGGGTAAATGTTGG | GCTTTGTCCCTGAAAGATGG |
| **3** | 192 | TGCAGGGATTATTATTAAGC | TCACTGAGAAGCAGATTTCCTC |
| **4** | 272 | ATGGCTGCACACAAATTCA | CCATGCCTGGCCTAAGATAA |
| **5** | 218 | TCACTCCTCTTGCAAACAGA | TCCATATTCTCTTGGTTGATCTGA |
| **6** | 184 | TGTTCTAAGGCTGCTTTTGT | GCACCCTGGACACTGTAATG |
| **7** | 221 | GCTCCCAATGGGCTAGAATC | AAGGCTAGATGAAAAGCAACCA |
| **8** | 235 | AAATAGATTTGGTGCCTATCCT | ACAAGGAAGCTGTACCTTAAAT |
| **9** | 195 | AGGTACAGCTTCCTTGTTGAA | TTCTTCTTATTGCCTCCTCTGG |
| **10** | 205 | CCCTTTTCCTTCCTTGTCCT | GGGAAATGACAATCAAGTTTCTTAC |
| **11** | 377 | (a) ACGGTGGAGAAGCAGTTGTC  (b) AGGATTACAGATGCTTTCCTCAA | (a) CCTCCAGCACCTGTAATCTCTATG  (b) CAATTCAGGGACTTCTTACAGTTTATC |
| **12** | 379 | (a) CATTTGCGAACTGCCTGTTA  (b) CCAAATTGAAAGACGCACGAGAG | (a) CGTCTCACCCACATACTTGA  (b) GGTCTCCTCTTGGAGGACAGA |
| **13** | 166 | TGTGGCTCAAAGACTTGATGA | TGGGTGCTTTATCTTTAAAACG |
| **14** | 296 | (a) TGCTCCTACAGCCCTGAGTT  (b) CAACATGGATTATGGAATGCAAGA | (a) GGCAGTCTTACAATAGAAGCTAACA  (b) CTATAAAGATTTGCTACATGCCACT |
| **15** | 353 | (a) GGACCAGCTGATATCTCCAATG  (b) CAGAGCAGCTGATTCGAGTA | (a) CTTGCGGCATACAAACTCTTTC  (b) AAATGGGAACTTTTCAGCAGAT |
| **16** | 308 | (a) CTCAGAAATAAGATGATGGAAACTGG  (b) CCCACTCATAACACCTCAAA | (a) CGGAGGCGAGTTGGATTT  (b) TGCAGGAGAGGGAGTTT |
